# PCOS Serum Promotes Endometrial Cancer Cell Proliferation with Partial Involvement of IGF-Associated AKT/mTOR Signaling: A Pilot Translational Study with Exploratory Molecular Insights

**DOI:** 10.3390/ijms27156594

**Published:** 2026-07-24

**Authors:** Neha Sharma, Mahmood Hachim, Syeda Sadaf Rizvi, Baila Samreen, Sumayya Inuwa, Tasneem AbuHajjaj, Fatima Ba Khamis, Fatma Alqutami, Aaron Han, Ibrahim Elrahman, Aparna Gumma, Uloma Okwuosa, Komal Hazari, Muna Tahlak, Fadi G. Mirza, William Atiomo

**Affiliations:** 1 College of Medicine, Mohammed Bin Rashid University of Medicine and Health Sciences, Dubai Health, Dubai 505055, United Arab Emirates; neha.sharma@dubaihealth.ae (N.S.); mahmood.almashhadani@dubaihealth.ae (M.H.); syedasadaf.rizvi@dubaihealth.ae (S.S.R.); baila_821@hotmail.com (B.S.); sumayya.inuwa@kch.ae (S.I.); fatma.alqutami@dubaihealth.ae (F.A.); aaron.han@lumc.edu (A.H.); matahlak@dubaihealth.ae (M.T.); fgmirza@dubaihealth.ae (F.G.M.); 2 Kings College Hospital London, Dubai Hills, Dubai 505055, United Arab Emirates; ibrahim.elrahman@kch.ae (I.E.); uloma.okwuosa@kch.ae (U.O.); 3 Latifa Hospital, Dubai Health, Dubai 505055, United Arab Emirates; taabuhajjaj@dubaihealth.ae (T.A.); fahbakhmais@dubaihealth.ae (F.B.K.); khazari@dubaihealth.ae (K.H.); 4 Department of Pathology, Stritch School of Medicine, Faculty of Health Sciences, Maywood Health Sciences Campus, Loyola University Medical Center Stritch School of Medicine, Chicago, IL 60153, USA; 5 Mediclinic Parkview Hospital, Dubai 505055, United Arab Emirates; aparna.gumma@mediclinic.ae

**Keywords:** polycystic ovary syndrome, insulin-like growth factor one, IGF1, endometrial, cancer

## Abstract

Women with polycystic ovary syndrome (PCOS) have a substantially increased risk of endometrial cancer (EC), yet the biological mechanisms underpinning this association, to support future prevention and therapeutics, remain incompletely understood. Insulin-like growth factor (IGF)-associated signaling has been implicated, but existing evidence is conflicting. In this pilot translational study, serum IGF1 and IGFBP-3 were measured in women with PCOS (*n* = 12 for IGF1 and *n* = 6 for IGFBP-3) and controls (*n* = 24 for IGF1 and *n* = 7 for IGFBP-3). Pooled serum, stratified by IGF bioactivity, was applied to human EC cells to further assess effects on cell viability, cell cycle distribution, and downstream signaling. Computational analysis of publicly available endometrial cancer datasets was used to contextualize experimental findings. Serum IGF1 and IGFBP-3 levels did not differ significantly between PCOS and control groups. However, pooled PCOS serum was associated with increased EC cell viability, altered cell cycle progression and PI3K/AKT/mTOR signaling compared with control serum in this exploratory model. Pharmacological inhibition of IGF1R partially attenuated these effects, suggesting that IGF-associated pathways may contribute but are unlikely to act in isolation. In silico analysis identified frequent alterations in PI3K/AKT/mTOR-related genes in EC, consistent with pathway-level vulnerability rather than IGF1-specific dependence. These findings suggest that PCOS serum contains factors that are associated with increased EC cell viability and altered signaling pathways with partial involvement of IGF signaling; however, these findings should be interpreted cautiously given the exploratory pooled-serum design, small subgroup sizes, and use of a single EC cell line. However, multiple metabolic and hormonal pathways are likely to contribute. Larger, better-controlled studies incorporating insulin, sex steroids, and multiple EC models are required before causal inferences can be made.

## 1. Introduction

Endometrial cancer (EC) is the second most diagnosed gynecological cancer in women globally, and its incidence has risen by 132% in the past 30 years [1,2]. Given this rising burden, understanding established risk factors such as polycystic ovary syndrome (PCOS), the most frequently diagnosed endocrine disorder in women, is of increasing clinical importance. While the exact cause of PCOS is not fully understood, it is believed to be a multifactorial disorder involving genetic, metabolic, endocrine, and environmental components. It can impact a woman’s life, manifesting in puberty with menstrual irregularities, and continuing through reproductive years, leading to infertility, obesity and insulin resistance [3]. Early diagnosis is crucial to minimize long-term complications like type 2 diabetes, hypertension, and EC. PCOS is specifically associated with a 3 to 5 times increase in the risk of EC [4,5,6]. Notably, many affected women are diagnosed at a young age and require hysterectomy, resulting in permanent sterility [7]. Despite this, effective screening and prevention strategies for EC remain lacking [8], unlike cervical cancer, which benefits from well-established protocols [9]. Major EC risk factors—besides PCOS—include obesity, diabetes, nulliparity, and chronic anovulation [10]. However, how to effectively translate these into a clinical screening tool remains uncertain. Given the global prevalence of PCOS, understanding how it increases EC risk could have a significant impact on public health.

Suggested mechanisms for the increased EC risk in PCOS include obesity, unopposed high estrogen levels, raised testosterone levels [11], anovulation and raised insulin-like growth factor-1 (IGF1) [12,13]. However, the extent to which these mechanisms increase EC risk or interact with each other to cause EC in PCOS is unknown [10]. New genes and proteins that might cause EC in PCOS as well as upregulate genes associated with the insulin signaling pathway in PCOS have also been identified [14]. Evidence suggests that insulin resistance and elevated IGF1 levels may play a key role in the association between PCOS and EC, as women with PCOS develop insulin resistance, and these factors have been linked to endometrial dysfunction and miscarriages [15]. Additionally, IGF1 and IGF2 have been shown to stimulate cell growth in human EC cell lines [16]. EC is broadly classified as Type I and Type II cancers. Type I EC is typically estrogen-dependent, endometrioid in histology, and associated with a favorable prognosis. In contrast, Type II EC is generally estrogen-independent, includes serous and clear cell histologies and is associated with a more aggressive disease and poorer outcomes [17]. Mutations in the PI3K pathway are common in Type I EC, and elevated levels of IGF1 and insulin can promote cell proliferation through this pathway. Other molecular pathways, such as the TP53 and ERK pathways, also contribute to EC progression. Dysfunction in the TP53 pathway is well known in Type II EC [18], while overactivation of ERK pathway can stimulate cell growth and migration [19]. These pathways interact with the IGF1 signaling pathway, and certain IGF1 gene promoter polymorphisms have been linked to EC [13]. However, the exact relationship between serum IGF1 and EC remains unclear, with conflicting results from different studies. Understanding the role of the IGF1 pathway in the association between EC and PCOS is crucial, as the PI3K pathway, which is often dysregulated in EC, is a key part of insulin and IGF1 signaling. It would also provide a stronger basis for the use of Metformin, a drug that targets the insulin signaling pathway, in the treatment of endometrial hyperplasia, a potentially premalignant condition common in PCOS [20].

In a previous study, serum and endometrial gene expression of IGF1 levels were raised in women with PCOS and EC compared with controls, but the women with EC had a higher body mass index and age [12], limiting interpretation of whether these differences were attributable to malignancy, metabolic factors, or age-related effects. Given the complexity of PCOS and the heterogeneity of EC, we designed this study as a feasibility and hypothesis-generating investigation. Our aim was not to establish causality but to explore whether serum from women with PCOS exerts differential biological effects on EC cells compared with controls, and whether IGF-associated signaling might contribute to these effects within a broader network of metabolic and hormonal influences. This approach moves beyond association to functional mechanism and provides a translational basis for improved risk stratification and targeted prevention and therapeutic strategies in the PCOS-EC clinical context.

## 2. Results

### 2.1. Participant Characteristics

PCOS participants were younger than controls and had similar BMI, although the BMI distribution differed from those reported in population-based PCOS cohorts. LH/FSH ratios were elevated in a subset of PCOS participants; some control participants also showed higher ratios, highlighting diagnostic overlap and real-world heterogeneity. Table 1 outlines the demographic characteristics of the women who participated in the study (N = 24 control and N = 12 PCOS). Women in the PCOS group were younger (27 vs. 35 years, *p* = 0.003), and 25% of them had had a previous pregnancy compared to 71% of women in the control group (*p* = 0.009). However, there was no significant difference in mean body mass index (BMI) or blood pressure. LH/FSH ratios were overall higher (>2) in the PCOS group, with six out of 11 women compared to the control group with two out of 15 women (*p* = 0.024). To minimize any bias between the control and PCOS groups, a clinically matched subgroup of women was selected for further analysis (N = 7 control and N = 6 PCOS). There was no statistically significant difference in age, BMI, blood pressure, and proportion of those with a previous pregnancy. The LH/FSH ratio was measured in three control women, none of whom exhibited a higher ratio compared to the PCOS group, where two out of six women showed an elevated LH/FSH ratio. Furthermore, based on their IGF1/IGFBP-3 ratios, both control and PCOS patients were categorized into four groups (Table 2). The details of the demographic characteristics of these patients are outlined in Table 2, and samples from these patients were used in subsequent experiments. No significant differences were observed in age, BMI, ethnicity, previous pregnancy history, or smoking and drinking habits across the groups. All the women in the PCOS group had a positive ovarian USS diagnosis. However, there was a significant difference in menstrual regularity; none of the women in the control group had irregular menstruation, while only one woman in the PCOS low IGF1/IGFBP-3 group had regular periods. Additionally, the control groups with low IGF1/IGFBP-3 had lower systolic blood pressure than the other groups. Testosterone levels were assessed in one control patient (Group B with Low IGF1/IGFBP-3), who had slightly elevated levels (1.9 nmol/L) compared to two out of six PCOS patients with high testosterone (each in PCOS group with low and high IGF1/IGFBP-3). One control patient had a small dermoid cyst. Among the PCOS group, one patient had asthma, and two had iron deficiency anemia, whereas only one control patient had anemia due to chronic blood loss.

### 2.2. Serum IGF1 and IGFBP-3

**Figure 1:**
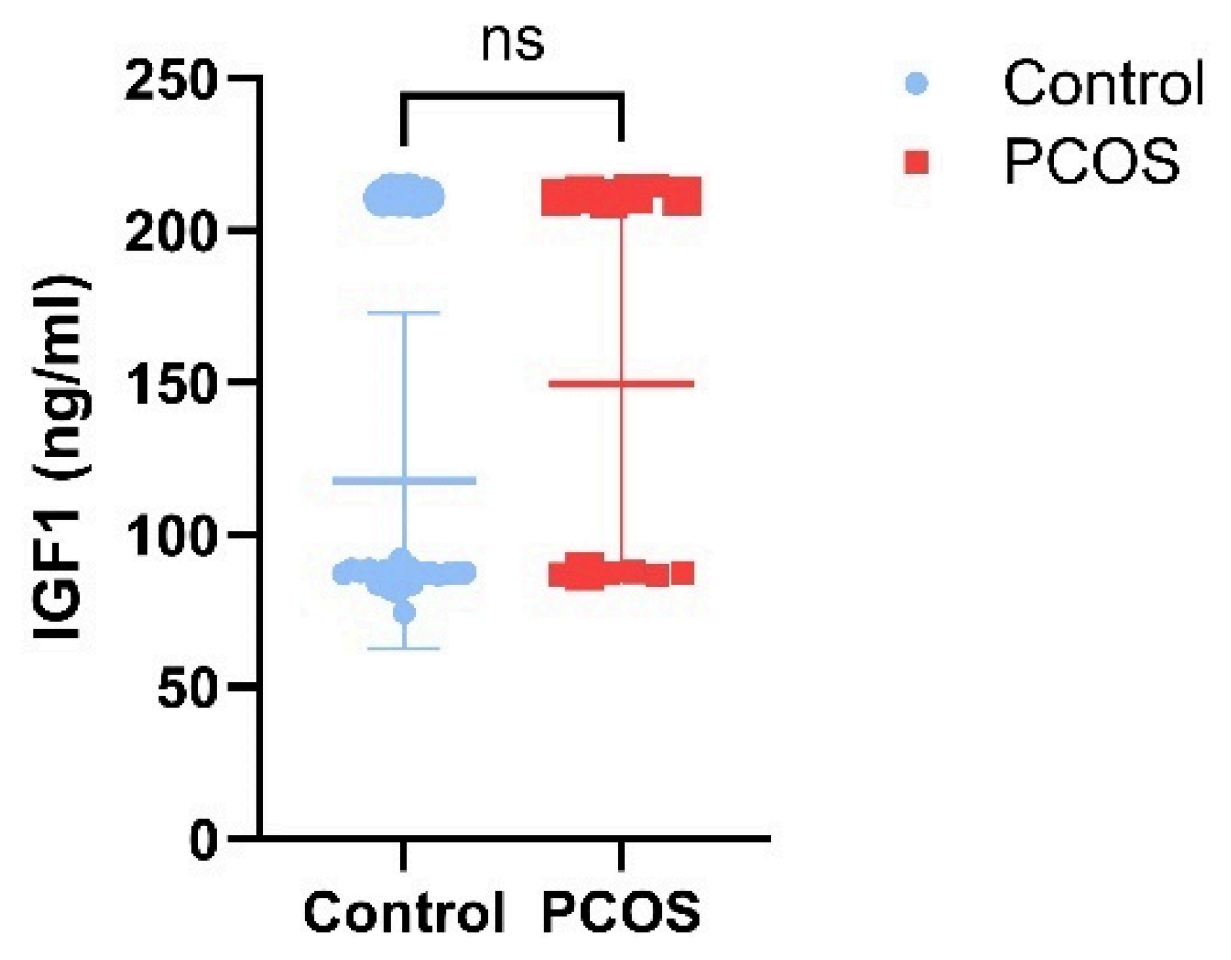
Quantification of Serum IGF1 levels. ELISA was performed to quantify IGF1 protein levels in the serum of Control (*n* = 24) and PCOS (*n* = 12) patients. Statistical significance was assessed using an unpaired *t*-test to compare mean differences (PCOS–Control), with *p*-values < 0.05 considered significant. Error bars represent the SD. Where ng/mL = nanograms per milliliter, ns = not statistically significant.

**Figure 2:**
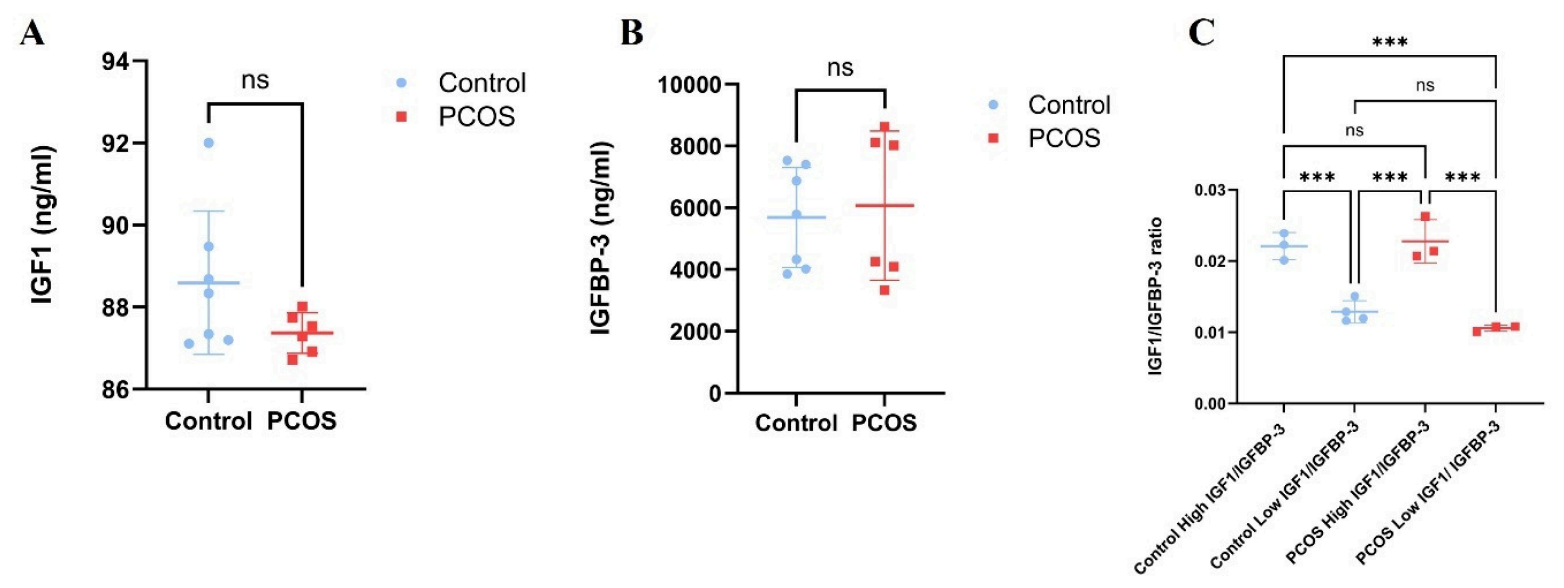
Quantification of Serum IGF1 and IGFBP-3 and stratification of Control and PCOS groups based on IGF1/IGFBP-3 ratio. ELISA was performed to quantify the levels of (**A**) IGF1 and (**B**) IGFBP-3 in Control (*n* = 7) and PCOS patients (*n* = 6). Statistical significance was calculated by using an unpaired *t*-test. (**C**) Serum IGF1/IGFBP-3 ratios were quantified into four different groups as Control High IGF1/IGFBP-3 (N = 3), Control Low IGF1/IGFBP-3 (N = 4), PCOS High IGF1/IGFBP-3 (N = 3) and PCOS Low IGF1/IGFBP-3 (N = 3) based on their significantly different IGF1/IGFBP-3 ratio. Statistical significance was calculated using one-way ANOVA followed by Tukey’s multiple comparison test, with *p*-values < 0.05 considered significant. Error bars represent SD. *** <0.001, ns—not statistically significant.

Serum IGF1 levels were analyzed in blood samples from 24 non-PCOS control women and 12 PCOS women. No statistically significant difference was observed between the two groups (117.8 ± 55.2 ng/mL vs. 149.6 ± 65.0 ng/mL, *p* = 0.13; Figure 1). Considerable inter-individual variability was observed, and subgroup analyses were strictly exploratory. A subset of clinically matched controls was selected (N = 6 PCOS, N = 7 control). Consistent with the broader dataset, IGF1 levels remained similar between these subgroups (88.6 ± 1.7 ng/mL vs. 87.4 ± 0.5 ng/mL, *p* = 0.12; Figure 2A). Mean IGFBP-3 levels did not differ significantly between control (5684 ± 1620 ng/mL) and PCOS groups (6072 ± 2417 ng/mL; *p* = 0.73; Figure 2B).

However, both groups contained patients with distinctly high or low IGFBP-3 levels, revealing significant intra-group variability (Control Low: 4067 ± 243.4 ng/mL, Control High: 6897 ± 793.7 ng/mL; PCOS Low: 3892 ± 496.2 ng/mL, PCOS High: 8251 ± 324.4 ng/mL; *p* = 0.001; Figure 2B, Table 2). Based on the IGF1/IGFBP-3 ratio, patients were stratified into four distinct subgroups: Control High Ratio (N = 3), Control Low Ratio (N = 4), PCOS High Ratio (N = 3), and PCOS Low Ratio (N = 3) (Table 2; Figure 2C).

### 2.3. PCOS Serum Enhances EC Cell Metabolic/Proliferative Activity and Alters Cell Cycle Progression, Partially via IGF-Associated Signaling

**Figure 3:**
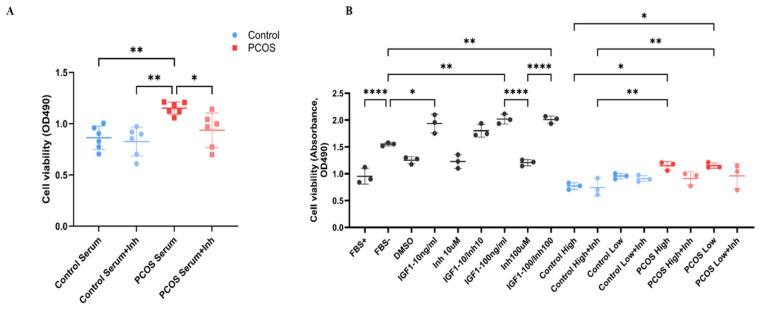
Effect of Control and PCOS serum on the proliferation of EC cells. The *X*-axis displays different treatment conditions over 24 h, and the *Y*-axis represents absorbance (at 490 nm), which correlates with cell viability or proliferation. (**A**) Direct comparison of the effect of Control and PCOS serum on EC cell viability/proliferation. (**B**) EC cells were treated with IGF1 (10 ng/mL and 100 ng/mL), IGF1R inhibitor (10 µM and 100 µM respectively), and IGF1 and IGF1R together with FBS (FBS+) or without FBS (FBS-) and DMSO as controls. Control and PCOS High represent groups with a High IGF1/IGFBP-3 ratio whereas Control and PCOS Low represent groups with a low IGF1/IGFBP-3 ratio. All groups were treated with 10 µM IGF1R inhibitor (Inh). Statistical significance was calculated by using one-way ANOVA followed by Tukey’s multiple comparison test, where *p* < 0.05 was considered significant, and the error bar represents the SD. **** <0.0001, ** <0.01 and * <0.05. ns—not statistically significant.

Pooled PCOS serum increased EC cell metabolic activity compared with control serum (*p* < 0.001; Figure 3A), suggesting perhaps an oncogenic influence of PCOS serum. Partial attenuation by IGF1R inhibition was observed in the PCOS group but not in controls, indicating that IGF-associated signaling may contribute, but is unlikely to be the sole mediator. Overall, PCOS serum induced greater EC cell viability than Control serum. This effect persisted under serum-deprived conditions, suggesting the presence of growth-promoting factors within PCOS serum. In addition, EC cells were treated with pooled patient serum from each subgroup (1:10 dilution) stratified by IGF1 bioactivity (Control High/Low and PCOS High/Low). Cells were also treated with exogenous IGF1 at 10 ng/mL (experimental equivalent) and 100 ng/mL (physiological equivalent), with or without IGF1R inhibitor (10 µM or 100 µM, respectively). Serum-free, FBS-supplemented, and DMSO controls were included (Figure 3B). IGF1 treatment increased EC cell viability, while IGF1R inhibition reduced it. Notably, co-treatment with IGF1 and IGF1R inhibitor did not further decrease viability beyond IGF1 treatment alone, consistent with partial pathway dependence.

**Figure 4:**
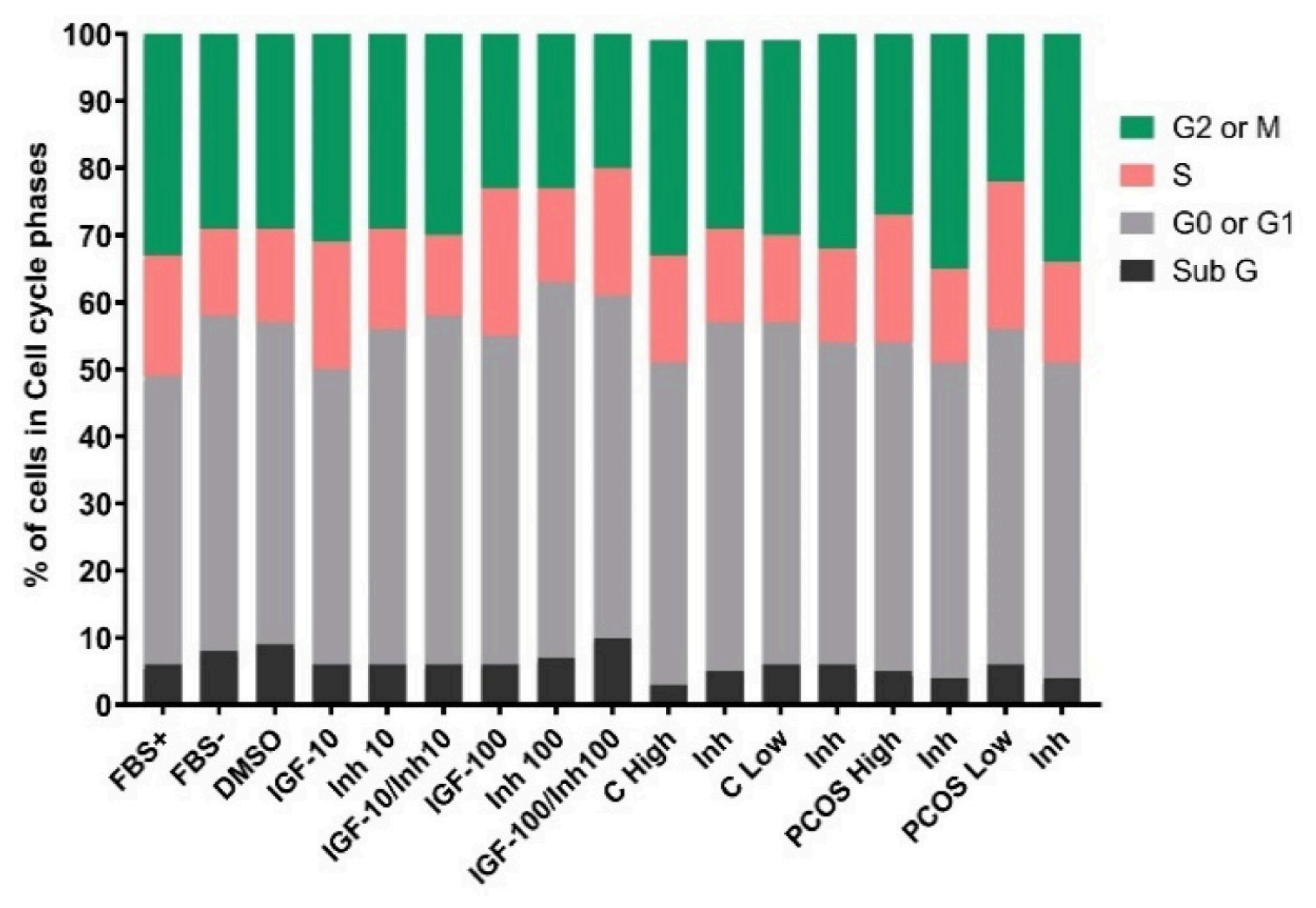
Effect of Control and PCOS serum treatment on cell cycle phases of EC cells. HEC1-A cells were treated with serum from control and PCOS groups in addition to IGF1 (10 ng/mL and 100 ng/mL), IGF1R inhibitor (10 µM and 100 µM respectively), and IGF1 and IGF1R together. Control and PCOS High represent groups with a high IGF1/IGFBP-3 ratio, whereas Control and PCOS Low groups represent a low IGF1/IGFBP-3 ratio. All groups were treated with 10 µM IGF1R inhibitor (Inh). EC cells with FBS (FBS+) or without FBS (FBS−) and DMSO were used as controls. EC cells were fixed and stained with PI-dye, counted based on their DNA content using a flow cytometer, and plotted in a graph representing the cell count in each condition. *X*-axis displays different treatment conditions over 24 h, and the *Y*-axis represents % of cells in cell cycle phases.

We further investigated the impact of PCOS serum on the cell cycle distribution across various treatment groups in EC cells (Figure 4). Cell cycle analysis revealed a normal distribution in EC cells treated with FBS+, with most cells in the G0/G1 growth phase. A slight increase in the G1 phase and a decrease in the S phase (DNA synthesis) were observed in cells treated with FBS- and DMSO, likely due to serum withdrawal. EC cells treated with IGF1 (IGF1-10 ng/mL and IGF1-100 ng/mL) showed a higher percentage of cells in the S phase, indicating that IGF1 may promote cell cycle progression. In contrast, cells treated with inhibitors (10 µM and 100 µM) exhibited a reduction in the S phase and a higher proportion of cells in the G0/G1 phase, suggesting a potential cell cycle arrest. In control serum groups, EC cells displayed a relatively uniform cell cycle distribution, with approximately 50% of cells in G0/G1, 14% in S, and 30% in G2/M, suggesting normal cell cycle progression. Both PCOS High and Low groups showed notable differences, with an increase in S phase (around 21% of the cells) and a decrease in G2/M phase (around 24%) compared to controls (Figure 4). This exploratory cell cycle analysis suggested increased S-phase representation in PCOS serum-treated cells. Blocking IGF1R reversed the PCOS-associated cell cycle shift, resulting in a decrease in S phase (around 14%) and a sharp increase in G2/M phase (around 34%) compared with controls.

### 2.4. Differential Modulation of AKT/GSK3β/mTOR Signaling Under PCOS Conditions

**Figure 5:**
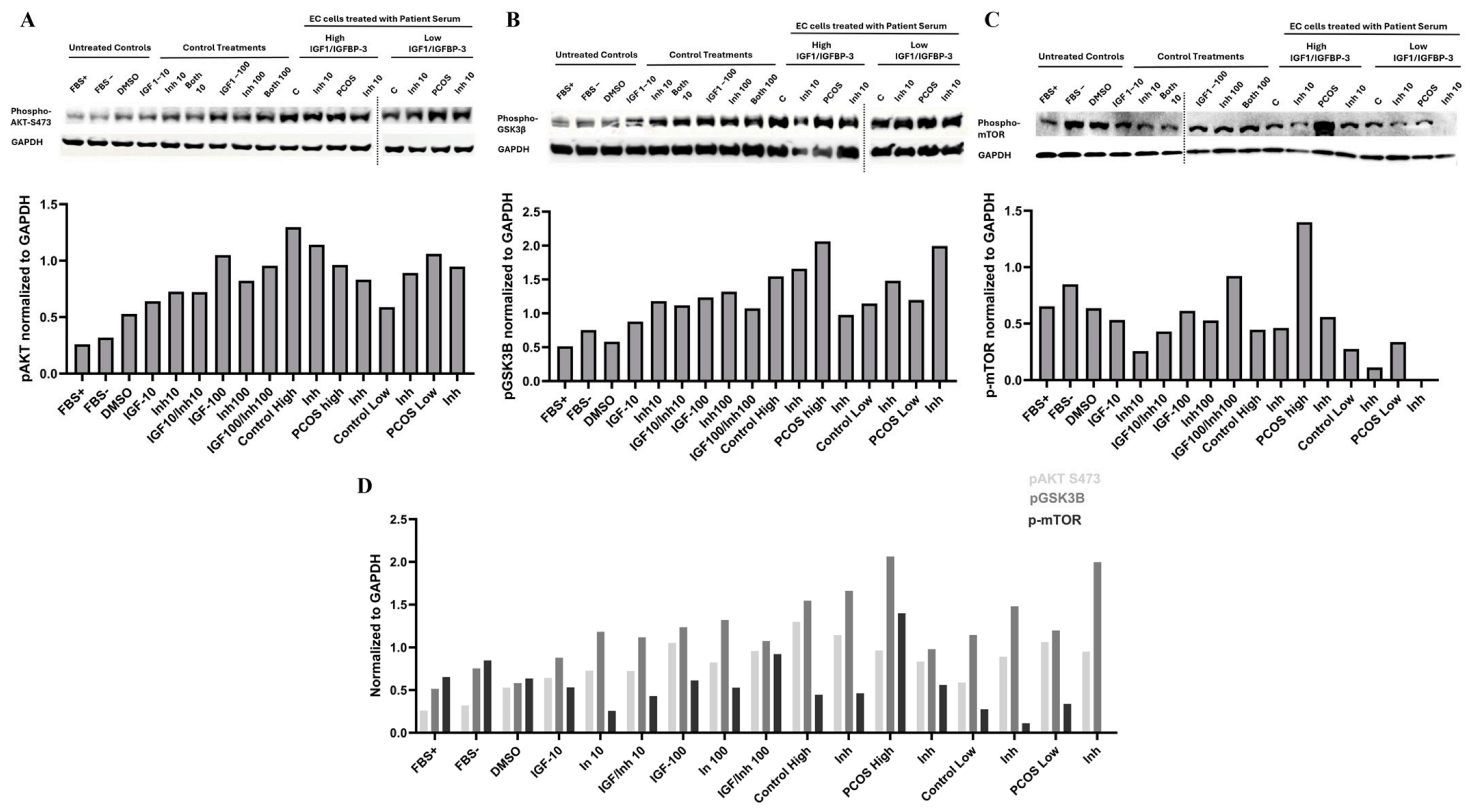
Protein quantification using Western blot. EC cells were treated with different treatments and serum from four different control and PCOS groups. EC cells with FBS (FBS+) or without FBS (FBS-) and DMSO were used as controls. Additionally, EC cells were treated with IGF1 (10 ng/mL and 100 ng/mL), IGF1R inhibitor (10 µM and 100 µM respectively), and IGF1 and IGF1R together (Both). Control and PCOS High represent groups with a high IGF1/IGFBP-3 ratio, whereas control and PCOS Low represent a low IGF1/IGFBP-3 ratio. All groups were treated with 10 µM IGF1R inhibitor (Inh). Proteins were immunoblotted for (**A**) pAKT, (**B**) pGSK3β and (**C**) p-mTOR. GAPDH was used as a loading control for each blot, and the protein levels were quantified after normalization to GAPDH. Bands after the delineated lines are merged from a separate blot run in parallel under identical conditions due to space limitations in capturing all samples on a single blot. Original blots are presented in Supplementary Figure S1. (**D**) Quantified protein levels of pAKT, pGSK3β and p-mTOR were combined and represented together.

Western blot analyses demonstrated variable activation of AKT, GSK3β, and mTOR, with inconsistent responses to IGF1R inhibition, underscoring the complexity of the pathway rather than a linear IGF1 dependence. IGF stimulation increased AKT phosphorylation in a dose-dependent manner with partial attenuation following IGF1R inhibition, confirming pathway engagement in this model (Figure 5A). Under serum-treated conditions, signaling responses were heterogenous and context dependent. Both control and PCOS groups with high IGF bioactivity exhibited increased phosphorylation of GSK3β (p-GSK3β) and mTOR (p-mTOR), suggesting enhanced IGF1 signaling (Figure 5B,C). Notably, PCOS serum with high IGF bioactivity induced particularly strong activation of p-GSK3β and p-mTOR relative to matched control serum (Figure 5B,C). Inhibition of IGF1 signaling led to a marked decrease in p-GSK3β in the PCOS High group (Figure 5B), but not in the Control High group. This supports a possible model in which PCOS serum enhances EC cell survival and growth via these pathways. IGF1R inhibition more consistently reduced p-GSK3β and p-mTOR levels in PCOS serum-treated cells than in control conditions, whereas p-AKT responses were variable. In low IGF bioactivity contexts, IGF1R inhibition produced divergent effects, including paradoxical increases in p-GSK3β, underscoring pathway crosstalk and compensatory signaling. Contrary to our expectation, PCOS High displayed lower levels of pAKT protein than the Control High group, while PCOS Low showed increased levels of pAKT than the control, suggesting differential activation of IGF1 signaling within PCOS subgroups. IGF1 inhibition moderately reduced p-AKT levels across all groups except Control Low, where inhibition paradoxically increased p-AKT. Collectively, these findings support a contributory, context-dependent role of IGF signaling in PCOS serum-driven EC cell growth rather than a linear or IGF-exclusive mechanism.

### 2.5. Genomic Alterations in the IGF1 Signaling Axis Identified Through In Silico Analysis

Genomic analysis demonstrated frequent alterations in PI3K/AKT/mTOR pathway genes across endometrial cancers, consistent with broad pathway activation by multiple upstream inputs. A large number of significant co-occurrences were observed between genes within the IGF1 pathway, suggesting that multiple nodes are frequently co-activated in endometrial tumors (Supplementary Table S2). Examples include: IGF1–IGF1R, IGF1R–IRS1/2, and IGF1–IRS1/2, highlighting simultaneous activation at the ligand–receptor–adaptor levels. Frequent co-occurrence between IGF1R and MAPK/PI3K-pathway components such as MAP2K1, RAF1, PIK3CB, and MTOR indicates dual pathway signaling in many tumors (Supplementary Tables S2 and S3). These combinations imply a cooperative signaling effect where enhanced growth and survival signals converge from both MAPK and PI3K/AKT axes, reinforcing the robustness of IGF-driven oncogenic activity. A significant mutual exclusivity between AKT1 and PTEN in EC was also identified, with tumors typically selecting one of these two pathways for AKT signaling activation—either by activating AKT1 or losing PTEN. This mutual exclusivity is due to redundant downstream effects.

**Figure 6:**
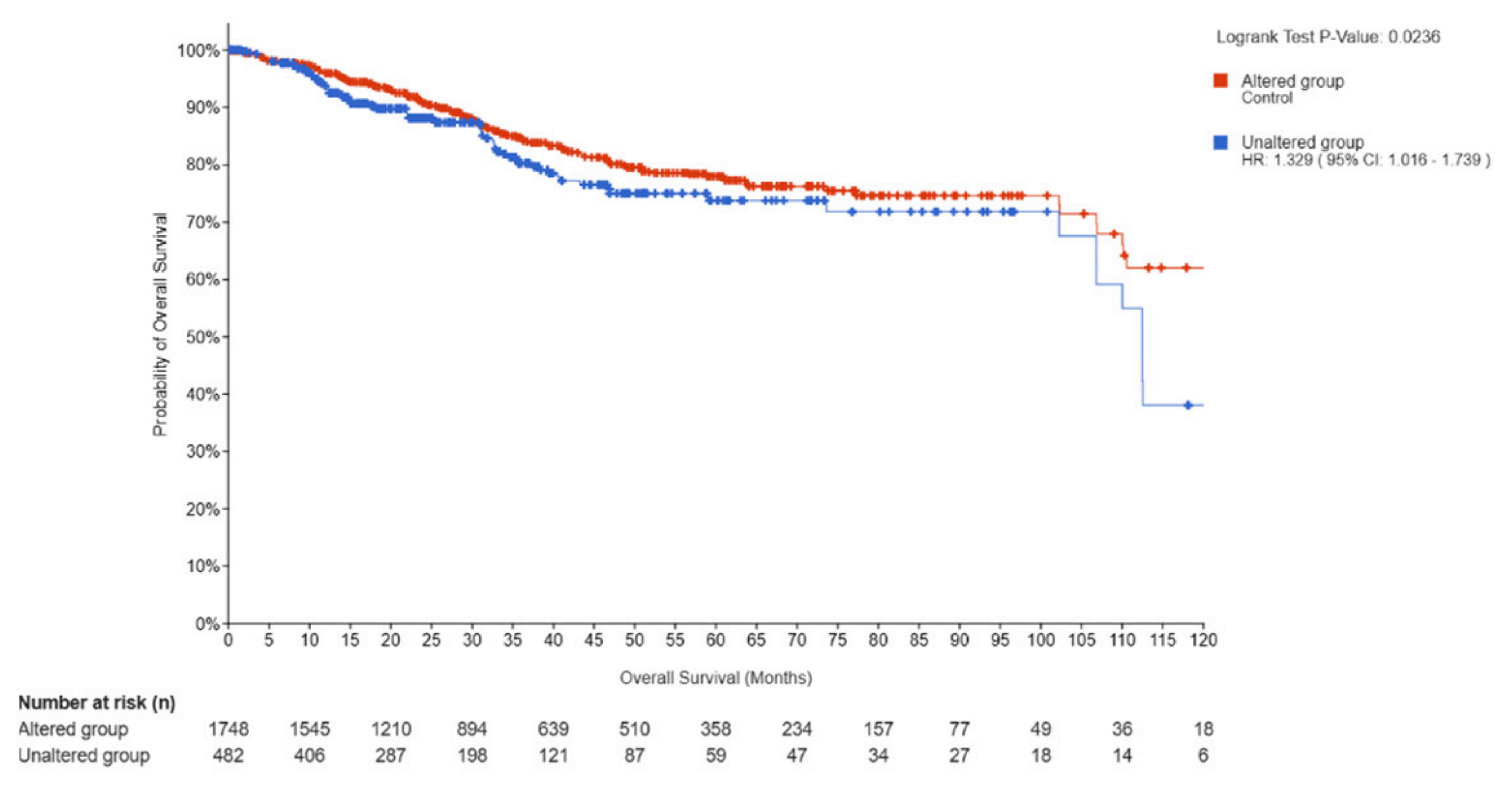
Survival analysis comparing patients with and without genetic alterations in key IGF signaling pathway genes.

A survival analysis (Figure 6) comparing patients with and without genetic alterations in key IGF signaling pathway genes revealed significant differences in overall survival (OS). Patients with tumors harboring at least one alteration in IGF pathway genes (such as IGF1, IGF1R, IRS1/2, and PIK3CA) had better OS than those without these alterations. The unaltered group had a 32.9% higher risk of death (Hazard Ratio = 1.329), and the difference was statistically significant (*p*-value = 0.0236). Further analysis of the clinical and molecular characteristics of IGF pathway-altered and unaltered tumors revealed several key trends. Tumors with IGF pathway alterations were enriched in genomically stable or hypermutated subtypes, such as POLE and MSI-H, and were more frequently of lower-grade histology (G1 and G2). In contrast, the unaltered group had a higher proportion of aggressive, poorly differentiated tumors (G3), often associated with serous histology. An investigation of the clinical and molecular feature distribution in IGF pathway-altered vs. unaltered groups also found that IGF-pathway alterations were enriched in endometrial tumors that were of a lower grade, endometrioid, MSI-H/POLE/CNV-low, and of a better prognosis molecular subtype.

## 3. Discussion

This pilot translational study sought to explore the feasibility of investigating the association between PCOS and EC risk through the lens of IGF1 signaling. Rather than testing a single definitive mechanism, our aim was to generate preliminary biological insights that may inform future, adequately powered studies examining the role of the IGF axis in PCOS-associated EC risk. By integrating clinical samples, targeted functional assays, pathway signaling, and in silico analyses, our findings suggest that PCOS serum can promote EC cell growth and signaling pathways in this exploratory model by partially engaging IGF signaling. Importantly, mean IGF1 levels were similar across the four subgroups, indicating that the observed biological effects cannot be explained solely by differences in total circulating IGF1 concentration. Instead, subgroup stratification was based on the IGF1/IGFBP-3 ratio as a surrogate marker of IGF bioactivity. While IGF1 concentrations showed minimal variation, substantial differences in IGFBP-3 levels were observed between groups, potentially influencing IGF1 bioavailability and downstream signaling. These findings suggest that dysregulation of the IGF system, rather than absolute IGF1 levels alone, may be more relevant to PCOS-associated EC biology. They also underscore the limitations of relying on mean serum levels in heterogeneous endocrine disorders, within a broader, disease-specific metabolic context. We observed no significant difference in circulating serum IGF1 levels between women with PCOS and controls. However, functional cell-based assays demonstrated that EC cell proliferation was higher in the pooled-serum exploratory assay in cells treated with PCOS serum compared to controls. While preliminary, this finding aligns with epidemiological data reporting a 3–5-fold increased risk of EC among women with PCOS, and supports the feasibility of using patient-derived serum to model disease-relevant biological effects in vitro in this context. Importantly, the absence of differences in total serum IGF1 suggests that IGF1 alone is unlikely to fully explain the observed proliferative effects, underscoring the need to consider regulatory components of the IGF system. In this context, IGFBP-3, a key modulator of IGF1 bioavailability and activity [21,22], emerged as a potentially important factor. As part of this pilot work, we therefore examined IGFBP-3 levels and stratified participants by IGF1/IGFBP-3 ratio as a surrogate for bioactive IGF1. Subgroup analyses are purely hypothesis-generating and require validation in larger cohorts. Although limited by small subgroup sizes, this stratification suggested that PCOS serum was associated with increased EC cell viability and proliferation across both high and low ratio subgroups relative to controls. Inter-individual variability within the control group likely influenced some of the unexpected findings, underscoring the challenges inherent in small pilot studies. For example, one control participant had mildly elevated testosterone levels, and another had a small dermoid cyst, both of which may alter IGF signaling. Additionally, slightly higher IGFBP-3 levels in one control sample may have disproportionately affected group-level proliferation outcomes. A key limitation of this study is the use of pooled-serum samples from small participant subgroups, which precluded assessment of within-group heterogeneity and may have masked participant-specific effects. Consequently, the pooled-serum findings should be interpreted as exploratory and hypothesis-generating, requiring validation in larger cohorts with individual-level serum analyses.

Testosterone has been reported to interact with the IGF system by modulating IGF1 signaling and influencing IGFBP expression. Experimental evidence suggests that testosterone and IGF interact to regulate IGFBP-3 production, with testosterone enhancing IGF-mediated increases in IGFBP-3 expression. This interaction may influence IGF bioavailability and downstream signaling, providing a potential mechanism through which hyperandrogenism could modulate IGF-associated effects in PCOS [23]. An observational and Mendelian randomization study identified positive associations between testosterone and endometrial cancer risk and suggested that the relationship between IGF and EC may be influenced by BMI. These findings further highlight the complex interplay between androgenic, metabolic, and IGF-related pathways in endometrial carcinogenesis [11]. Together with prior reports of IGF1 and IGFBP imbalance in PCOS pathophysiology [24], our findings support the feasibility and potential value of assessing IGFBPs alongside IGF1 in future mechanistic studies.

To further explore whether IGF1 signaling contributes to the observed biological effects, we employed pharmacologic inhibition of the IGF1 receptor (IGF1R). Although reductions in proliferation did not reach statistical significance consistent with the exploratory nature and limited power of this pilot study, a reproducible trend toward decreased EC cell viability was observed in PCOS serum-treated cells following IGF1R inhibition. Pooled-serum analyses supported this observation, showing that PCOS serum was associated with increased EC cell viability, which was partially attenuated by IGF1R blockade. These findings suggest that IGF1 signaling may contribute to but does not solely drive PCOS-associated EC cell proliferation. Cell cycle analyses further supported a contributory role for IGF1 signaling, with PCOS serum increasing the proportion of cells in S phase and decreasing those in G2/M phase—changes that were reversed by IGF1R inhibition. This pattern is consistent with IGF1-mediated cell cycle progression via PI3K and/or MAPK pathways, as reported in other cellular models [25,26,27]. While preliminary, these findings indicate that IGF1R inhibition can partially restore cell cycle regulation in this model system.

To generate mechanistic hypotheses, we examined downstream components of the AKT/GSK3β/mTOR signaling axis in EC cells treated with PCOS and control sera. Both PCOS and control groups with high IGF1/IGFBP-3 ratios demonstrated increased phosphorylation of GSK3β and mTOR, consistent with increased IGF1 bioavailability. Unexpectedly, p-AKT levels were higher in the Control High-ratio group than in the PCOS counterpart. Although this contrasts with reports of increased endometrial p-AKT expression in PCOS [28], it may likely reflect biological heterogeneity, small sample size, and the exploratory nature of this study. Additional possible explanations include negative feedback mechanisms within the PI3K/AKT/mTOR pathway, whereby chronic pathway stimulation attenuates downstream AKT phosphorylation [29]. Alternatively, differences in IGF bioavailability, IGFBP-mediated regulation, or other metabolic and hormonal factors present in PCOS serum may alter signaling dynamics independently of total IGF activity. Moreover, compensatory signaling through parallel pathways such as MAPK/ERK has been reported in response to perturbations in PI3K/AKT signaling, highlighting the complexity of pathway regulation in cancer cells [30]. This finding highlights the complexity of IGF-associated signaling in PCOS and suggests that pathway activation may not occur in a linear manner across all biological contexts.

In contrast, PCOS serum with a high IGF1/IGFBP-3 ratio induced the strongest activation of p-GSK3β and p-mTOR, supporting enhanced proliferative signaling. IGF1R inhibition suppressed these signals in PCOS-treated cells but not in controls, suggesting partial IGF1 dependence in PCOS. In the PCOS Low subgroup, IGF1R inhibition reduced p-mTOR but paradoxically increased p-GSK3β, potentially reflecting compensatory activation of alternate pathways such as MAPK and highlighting complex crosstalk between AKT-mTOR and GSK3β-mTOR signaling [31,32]. These findings suggest that metabolic factors common in PCOS, including insulin resistance and hyperinsulinemia, may modulate pathway activation independently of IGF1 signaling. Given the established roles of GSK3β and mTOR in metabolism, cell survival, and oncogenesis [33,34,35,36,37], these preliminary observations underscore the heterogeneity of PCOS phenotypes and suggest that IGF1R inhibition alone may be insufficient in certain metabolic contexts. Although IGF1R inhibitor was used to inhibit IGF1R signaling, the contribution of potential off-target effects cannot be completely excluded. Therefore, the findings should be interpreted as supporting involvement of IGF-associated signaling rather than definitive proof of IGF1R-specific causality. Future studies should incorporate more selective pharmacological or genetic approaches to distinguish IGF1R-dependent from insulin receptor-dependent effects and should assess insulin-related factors to better define the relative contribution of these pathways in PCOS-associated EC. Furthermore, validation in additional EC cell lines with different molecular backgrounds, such as Ishikawa and AN3CA, would help determine whether IGF-associated signaling plays a broader role across molecularly distinct endometrial cancer models.

To contextualize our experimental findings, we performed an in silico analysis of publicly available genomic datasets, which demonstrated a high frequency of mutations in the PI3K/AKT/mTOR pathway—particularly PTEN and PIK3CA—in EC. These findings support the biological relevance of the pathways examined experimentally and may explain some of the paradoxical signaling responses observed in vitro. Notably, improved overall survival in IGF-altered tumors observed in our in silico analysis is consistent with the predominance of less aggressive, Type I EC frequently associated with PCOS [38]. Taken together, these computational and experimental data suggest that altered IGF1 signaling may contribute to EC cell growth in a context-dependent manner and that PCOS may modulate this pathway in complex and heterogeneous ways.

The principal strength of this study lies in its translational approach, bridging clinical serum profiles with functional and molecular EC cell assays—an approach that complements prior epidemiological studies. However, as a pilot feasibility study, our findings are limited by a small sample size and inter-individual variability and should be interpreted as exploratory rather than definitive. These results nonetheless demonstrate the feasibility of this experimental approach and provide a framework for larger, well-powered studies incorporating detailed metabolic phenotyping and individual-level serum analyses to more definitively define the role of IGF1 dysregulation in PCOS-associated EC risk. All functional findings should therefore be regarded as hypothesis-generating and requiring validation in independent patient cohorts and additional endometrial cancer models.

Future studies should investigate MAPK/ERK signaling in PCOS-associated endometrial cancer, given its association and co-occurrences with IGF-related genes in our in silico analyses. Investigating crosstalk between these pathways may help explain the heterogeneous signaling responses observed across PCOS serum subgroups and provide a more comprehensive understanding of the molecular mechanisms linking PCOS and endometrial cancer. Addressing this complexity is particularly urgent given recent findings [39] identifying ITGA2 and ITGA3, both part of the IGF1/PI3K-AKT signaling pathway, as critical players in PCOS and EC pathology. Notably, our previously published in silico study [40] linked these integrins to PCOS and EC risk, suggesting that integrin inhibitors and metformin could be promising therapeutic avenues.

## 4. Materials and Methods

The methodological approach to this study has previously been published in a protocol paper [41] (International Registered Report Identifier (IRRID) DERR1-10.2196/48127).

### 4.1. Study Design and Participant Recruitment

This was a cross-sectional study at Kings College Hospital London, Dubai Hills and Latifa Women and Children’s Hospital, Dubai, with 12 women with PCOS and 24 women without PCOS (controls). The sample size was based on feasibility, recruitment logistics, the availability of eligible participants, and the exploratory translational design of the study. Physicians were requested to refer women with PCOS for inclusion in the study if they had a diagnosis of PCOS based on the Rotterdam Criteria [42], which include two or more of the following: clinical or biochemical evidence of hyperandrogenism, chronic oligo or anovulation, and polycystic ovaries on ultrasound with other causes of chronic oligo or anovulation excluded (e.g., hyperprolactinemia and hyperthyroidism). Inclusion criteria for controls were regularly menstruating women without a diagnosis of polycystic ovaries on ultrasound scan. Women identified as PCOS, or control were identified by gynecologists and residents in participating hospitals and provided a patient information sheet. The principal investigator of the study or one of the research assistants based in the laboratory at Mohamed Bin Rashid University (MBRU), Dubai, was then informed by the clinician (gynecologist or resident) who had identified a suitable participant for inclusion in the study, and arrangements were made for the participant to come into the hospital for a blood test following written consent. Informed consent was obtained from all the patients. With the permission of the participants, we also reviewed their electronic medical records (EMRs) to obtain details on their medical and gynecological history, including the reason for clinical presentation, menstrual history, date of last menstrual period before sample collection, past medical/surgical history, medication, allergies, and smoking history. The most recent height, weight, and blood pressure in the EMR was recorded, and the details of their last pelvic ultrasound scan were also recorded to confirm if their ovaries did or did not look polycystic. Hormonal assays, including LH and FSH, were not available for all participants due to real-world clinical testing constraints; denominators are therefore reported where applicable. This reflects routine clinical practice and is acknowledged as a limitation of this feasibility study. Women with known malignancy were excluded. However, minor gynecological comorbidities (e.g., small benign ovarian cysts, anemia) were not excluded in order to capture the clinical heterogeneity of PCOS; their potential influence is addressed in the Discussion. This study was explicitly designed as a pilot feasibility investigation; therefore, it is not powered for inferential clinical comparisons but to establish biological signal, variability, and methodological viability. We retained real-world heterogeneity to reflect clinical PCOS populations; matched subgroup analyses were performed to partially mitigate confounding, and consistent biological effects were observed.

### 4.2. Blood Samples; Processing and Storage

Fasting blood samples were obtained from the participants after pregnancy was excluded. Where possible, samples from control participants were obtained in the early follicular phase, while PCOS samples were collected opportunistically due to cycle irregularity. Menstrual cycle phase may represent an additional source of biological variability. Among participants included in the pooled-serum analyses, 3/7 control participants were sampled during the early follicular phase compared with 1/6 participants in the PCOS group. Estradiol, insulin, and glucose were not systematically measured and therefore could not be controlled for, which is a limitation in this study. The sample of blood was obtained from the participant’s arm and collected into one vacutainer for the study. Samples were transported from the hospitals to the laboratory at MBRU. Within 6 h of whole blood collection, tubes in specimen bags were transported on dry ice at temperatures between 2 and 8 °C. Whole blood was allowed to clot either for 15–30 min at room temperature or left at 4 °C for up to 24 h. After clotting, the blood was centrifuged at 1000× *g* for 10 min in a refrigerated centrifuge at 4 °C to separate the serum. The serum was carefully extracted with a fine-bore pipette to prevent red cell contamination and transferred to a sterile vial in an aseptic manner. Samples were kept at 2–8 °C during handling and stored at −80 °C in 0.5 mL aliquots until thawed for analysis.

### 4.3. Enzyme-Linked Immunosorbent Assay (ELISA) for Serum IGF1and IGFBP-3 Levels

Serum IGF1 (12 women with PCOS and 24 controls) and IGFBP-3 (6 women with PCOS and 7 controls) were measured using validated ELISA kits within the manufacturer’s detectable range. Extreme values were retained to reflect biological variability; distributions are described in the Results rather than relying solely on mean comparisons. The Human IGF1 ELISA Kit (ab100545; Abcam, Cambridge, UK) and Human IGFBP-3 ELISA Kit (ab211652; Abcam, Cambridge, UK) were used according to the manufacturer’s protocol to determine the IGF1 and IGFBP-3 levels in serum. The serum samples were diluted with the sample diluent (1:2 for IGF1 and 1:1500 for IGFBP-3), incubated with primary antibodies specific for IGF1 and IGFBP-3 protein, followed by the secondary antibody conjugated with HRP. The reaction was developed using TMB substrate, the plate was read at 450 nm, and the mean value was calculated from the triplicate reactions.

### 4.4. MTS Cell Viability/Proliferation Assay

HEC1A cells were used as a well-characterized EC model harboring PI3K pathway alteration. Cell viability was assessed using MTS assays as a screening tool, recognizing that this reflects metabolic activity rather than direct cell counts. Cell cycle analysis and Western blotting were used as supportive exploratory endpoints rather than definitive quantitative measures. IGF1R inhibition was employed as a pharmacological probe; given known cross-reactivity with insulin receptors, findings were interpreted cautiously as pathway-associated rather than IGF1-specific.

The human endometrial cancer cell lines (HEC1A cells) were maintained in Dulbecco’s Modified Eagle Medium (DMEM) containing 10% FBS and 1% penicillin-streptomycin. A total of 18,000 cells/well were seeded in a 96-well flat bottom plate and incubated for 24 h at 37 °C followed by serum starvation for 2 h prior to the treatment of EC cells with different treatment conditions performed in triplicate. EC cells were treated exogenously with IGF1 (ProspecBio CYT-216) alone (10 ng/mL and 100 ng/mL) or IGF1R inhibitor (ab141077) alone (10 uM and 100 uM) or co-treated with both IGF1 and IGF1R inhibitor (10 ng/mL and 10 uM; 100 ng/mL and 100 uM, respectively) to mimic the effect of patient’s physiological and experimental levels of IGF1 on EC cells. EC cells with or without serum media and DMSO (matched drug solvent) were used as controls. Pooled patient serum from different PCOS and control groups were diluted 1:10 and added to the EC cells alone or with 10 uM IGF1R inhibitor. Serum pooling was used to reduce inter-individual noise and enable detection of consistent biological effects at the group level, consistent with pilot feasibility-stage experimental design. However, individual-level serum analyses will be essential in future studies to identify patient-specific drivers. After 24 h, 20 uL/well MTS reagent (ab197010, Abcam, Cambridge, UK) was added to each well, and the plate was incubated for 3 h at 37 °C. The optical density was measured on a Luminex Microplate reader (Tecan, Mannedorf, Switzerland) at 490 nm according to the manufacturer’s protocol to quantify cell proliferation.

### 4.5. Cell Cycle Analysis via Flow Cytometry

Cell cycle was evaluated using a Propidium Iodide (PI) Flow kit (ab139418; Abcam, Cambridge, UK) according to the manufacturer’s protocol. HEC1A cells (7 × 10^5^ cells/condition) were seeded onto 6 wells and were incubated for 24 h in DMEM/10%FBS followed by serum starvation in serum-free media for 2 h prior to adding different treatment conditions to the EC cells, as previously described. After 24 h treatment, cells were trypsinized, centrifuged at 1500 rpm for 5 min and washed with 1× cold PBS. Subsequently, cells were fixed by adding ice-cold ethanol (70%) dropwise and incubated at 4 °C. Post fixation, cells were washed with 1× PBS and resuspended into 200 µL 1× PI solution for 30 min at 37 °C in the dark to enable staining. Before quantifying PI-stained cells by flow cytometry, the cells were counted via trypan blue. Analysis was conducted using Amnis^®^ CellStream^®^ Flow Cytometer (Luminex Corporation, Austin, TX, USA). Flow cytometry data were collected and analyzed using CellStream^®^ Analysis 1.3.384 software.

### 4.6. Western Blot

HEC1A cells (2.25 × 10^6^ cells/condition) were cultured on a 10 cm dish containing DMEM supplemented with 10% FBS for 24 h. Next day, cells were starved for 2 h in serum-free media prior to adding different treatments as previously mentioned and incubated for 24 h. Cells were washed and harvested in ice-cold PBS for protein extraction using 1× Lysis buffer (Cell Signaling Technology, Danvers, MA, USA) containing Protease and Phosphatase inhibitor cocktail (cat# 78441, Thermo Fisher Scientific, Waltham, MA, USA). Cell suspensions were sonicated to lyse, centrifuged at 14,000 rpm for 10 min at 4 °C, and supernatants were collected. Protein concentration was determined by Pierce BCA protein assay (cat# 23227 Thermo Fisher Scientific, Waltham, MA, USA). A total of 30 µg protein was separated on 10% SDS-polyacrylamide gel electrophoresis (SDS-PAGE) for pAKT and pGSK3β, and 8% SDS-PAGE gel for p-mTOR, to facilitate resolution of higher molecular weight targets. The gels were transferred to a nitrocellulose membrane using the Trans Blot Turbo Transfer system (Bio-Rad laboratories, Hercules, CA, USA). The membranes were blocked for 2 h at room temperature using 5% BSA in Tris buffer saline (TBS) with Tween 20 (0.1%) and incubated with primary antibodies against Phospho-GSK-3β (Ser9) (1:1000); Phospho AKT (Ser473) (1:1500); and phosphor-mTOR (1:700) (Cell Signaling Technology, Danvers, MA, USA) at 4 °C overnight. The membranes were washed with TBS-T three times for 30 min each at room temperature, followed by a 1-h incubation with Horseradish peroxidase (HRP)-conjugated secondary antibodies (1:3000). After washing, the membranes were developed using the enhanced chemiluminescence (ECL) Western blotting substrate kit (Cat# 1705061, Bio-Rad Laboratories, Hercules, CA, USA) as per the manufacturer’s instructions, and images were taken with the Azure Biosystems System. Glyceraldehyde 3-phosphate dehydrogenase (GAPDH) was used as the loading control. ImageJ software (NIH) version 1.54p was used to quantify the protein levels on Western blot. Due to the number of experimental conditions, samples were analyzed across two SDS-PAGE gels. To minimize inter-gel variability, all gels were prepared using the same gel composition and processed under identical electrophoresis, transfer, antibody incubation, and imaging conditions. Full uncropped blots with molecular weight markers are provided in Supplementary Figure S1.

### 4.7. In Silico Study of Publicly Available Genomic Data to Capture the IGF1 Signaling Axis

Publicly available EC datasets were interrogated to assess the frequency and co-occurrence of alterations in IGF-associated and downstream signaling pathways. This analysis was intended to contextualize experimental findings rather than establish prognostic relevance. Genomic data was retrieved from cBioPortal, cancer genomics database (https://www.cbioportal.org/) accessed on 25 April 2025, combining 17 datasets from MSK, TCGA, CPTAC, and other consortia, which included samples from various cancer subtypes such as endometrioid, serous, carcinosarcoma, and clear cell. A complete list of the 17 cBioPortal datasets included in the analysis is provided in the Supplementary Table S1. To focus on EC, a subtype filter identified 4076 patients, including those with endometrial carcinoma and uterine endometrioid carcinoma. For survival analyses, gene alteration status was defined using cBioPortal composite genomic profiles, including somatic mutations, copy number alterations (amplifications and deep deletions), and mRNA expression alterations, where available. Patients were classified as altered if at least one selected IGF-pathway gene exhibited any of these genomic alterations. A curated gene panel of 30 genes was selected to capture the major components of the IGF signaling network, including IGF ligands and receptors, IGF-binding proteins, adaptor proteins, and canonical downstream PI3K/AKT/mTOR and MAPK pathway effectors. Alterations were found in 80% of the queried patients, and co-occurrence and mutual exclusivity analyses were conducted using Fisher’s exact test and Benjamini–Hochberg FDR correction, with significant results visualized as a symmetric matrix.

### 4.8. Statistical Analysis

For the demographic data, continuous variables were summarized as means and standard deviations (SDs), and discrete variables were summarized as percentages. Statistical comparisons were performed using appropriate methods (e.g., *t*-tests for continuous variables and chi-square tests for discrete variables). For the ELISA assay, statistical significance was assessed using an unpaired *t*-test to compare mean differences (PCOS-Control ± SD), with *p*-values < 0.05 considered significant. Error bars represent the SD. For comparing 3 or more groups (MTS assay and different control and PCOS serum groups), statistical significance was calculated by using one-way ANOVA test followed by Tukey’s multiple comparison test, where a *p*-value less than 0.05 was considered significant. Although Tukey’s multiple comparison test was applied to account for multiple testing, the exploratory nature of this pilot study and the number of treatment comparisons performed warrant cautious interpretation of the statistical findings. For the MTS, cell cycle, and Western blot experiments, each treatment condition was performed using pooled-serum samples representing a single biological replicate (n = 3 participants per pool). Independent biological replicates were not feasible due to limited serum availability. Consequently, these findings should be interpreted as exploratory and hypothesis-generating. As this was a pilot study, no baseline sample size calculations were made. Experiments were conducted in triplicate, and the values are presented as mean ± SD. We recognize that opinions differ about the ideal sample size for cell culture experiments [43]. Given the exploratory nature of this study, statistical analyses were interpreted descriptively, with emphasis on consistent biological trends across assays rather than on isolated *p*-values.

## Figures and Tables

**Table 1:** Demographic characteristics of the women who participated in the study. PCOS = polycystic ovary syndrome, SD = standard deviation, BMI = body mass index, SBP = systolic blood pressure, DBP = diastolic blood pressure.

	Controls (*n* = 24)	PCOS (*n* = 12)	*p* Value
Mean age in years (SD)	35 (8)	27 (6)	0.0034
Number of women with previous pregnancy (%)	17 (71%)	3 (25%)	0.009
Mean BMI (SD)	27.4 (6.5)	24.5 (4.3)	0.18
Mean SBP (SD)	111 (11)	115 (9)	0.26
Mean DBP (SD)	72 (6)	71 (8)	0.57
Number of women with high LH/FSH ratio >2	2/15	6/11	0.024
Serum IGF1 level (ng/mL)	117.8 (55)	149.6 (65)	0.13

**Table 2:** Demographic characteristics of the women stratified by IGF1/IGFBP-3 ratio in both control and PCOS groups. PCOS = polycystic ovary syndrome, SD = standard deviation, BMI = body mass index, SBP = systolic blood pressure, DBP = diastolic blood pressure.

	Group A—Control (High IGF1/IGFBP-3) N = 3 Mean +/− SD	Group B—Control (Low IGF1/IGFBP-3) N = 4 Mean +/− SD	Group C—PCOS (High IGF1/IGFBP-3) N = 3 Mean +/− SD	Group D—PCOS (Low IGF1/IGFBP-3) N = 3 Mean +/− SD	ANOVA *p* Value
Age (years)	33 (8.1)	31 (9.4)	29.3 (10.0)	23.3 (1.5)	0.5
BMI	26.3 (2.3)	21.13 (3.9)	22 (5.5)	27.31 (5.3)	0.26
Number of women with UAE ethnicity (%)	3 (100%)	3 (75%)	3 (100%)	2 (67%)	NS
Number of women with previous pregnancy (%)	2 (33.3%)	2 (50%)	2 (33.3%)	3 (100%)	2.04 NS
Number of women with regular menstrual cycle	3 (100%)	4 (100%)	0 (0%)	1 (33.3%)	* *p* < 0.05
Systolic BP	116.7 (5.7)	95.2 (6.4)	124.3 (6.3)	112.3 (13.2)	0.007 ***
Diastolic BP	74 (0)	64.2 (1.7)	64 (9)	75.3 (11.0)	0.11
IGF1 (ng/mL)	89.53 (2.4)	87.89 (0.7)	87.61 (0.36)	87.13 (0.54)	0.1
IGFBP-3 (ng/mL)	4067 (243.4)	6897 (793.7)	3892 (496.2)	8251 (324.4)	<0.001 ****
IGF1/IGFBP-3	0.02 (0.0018)	0.01 (0.0015)	0.022 (0.003)	0.01 (0.0004)	0.001 ****

**** < 0.0001, *** < 0.001, * < 0.05 and NS-not significant.

## Data Availability

Data supporting the results reported in the article will be made available by the authors on request. cBioPortal data are publicly available. All queries can be directed to the corresponding author William Atiomo.

## Supplementary Materials

The following supporting information can be downloaded at https://www.mdpi.com/article/10.3390/ijms27156594/s1.
