# Supplementary material for: PCOS Serum Promotes Endometrial Cancer Cell Proliferation with Partial Involvement of IGF-Associated AKT/mTOR Signaling: A Pilot Translational Study with Exploratory Molecular Insights
Source: Int J Mol Sci. 2026 Jul 24;27(15):6594. doi: 10.3390/ijms27156594 (PMC13466320; doi:10.3390/ijms27156594)
Supplement: Supplementary file 1 [file ijms-27-06594-s001.zip › ijms-4309926-figures.pdf]

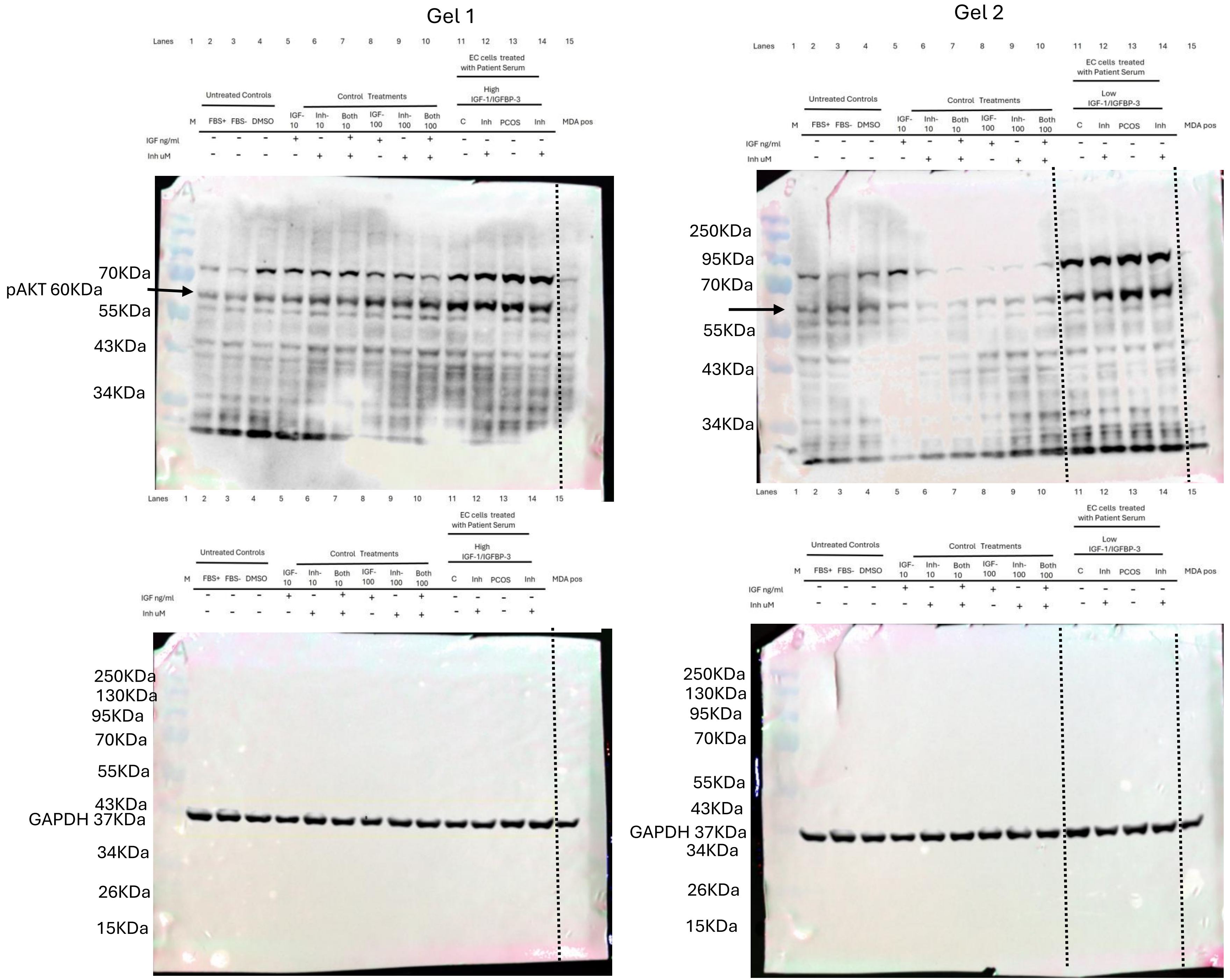

Figure S1 A) original pAKT and GAPDH western blots. Dash lines indicate the area From Gel2 cropped and merged with Gel1. Lane 1 is Marker and lane 15 is MDA cell protein lysate.

Dubai Health - Internal Use

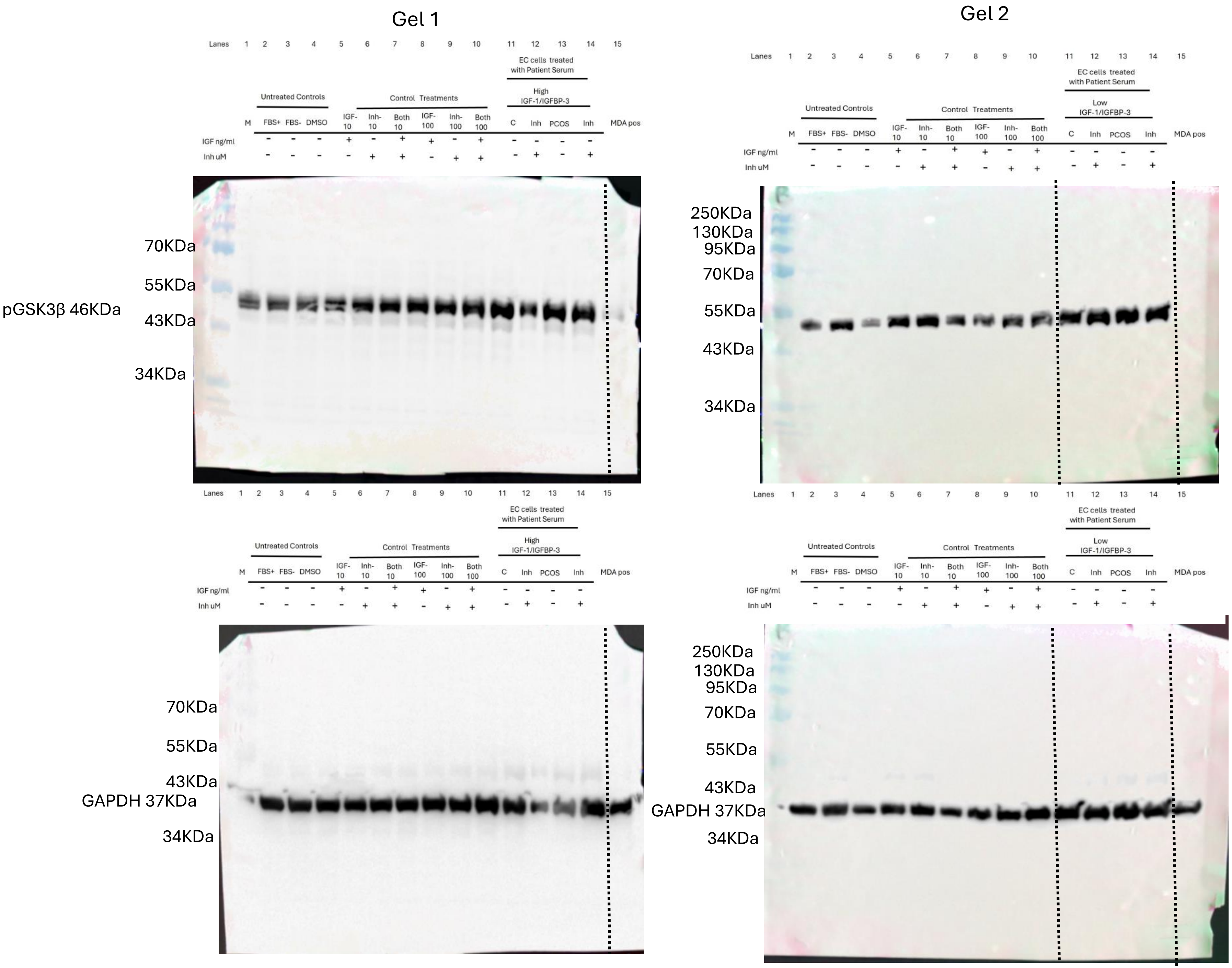

Figure S1 B) original 10% gel for pGSK3β and GAPDH western blots. Dash lines indicate the area From Gel2 cropped and merged with Gel1. Lane 1 is Marker and lane 15 is MDA cell protein lysate.

Dubai Health - Internal Use

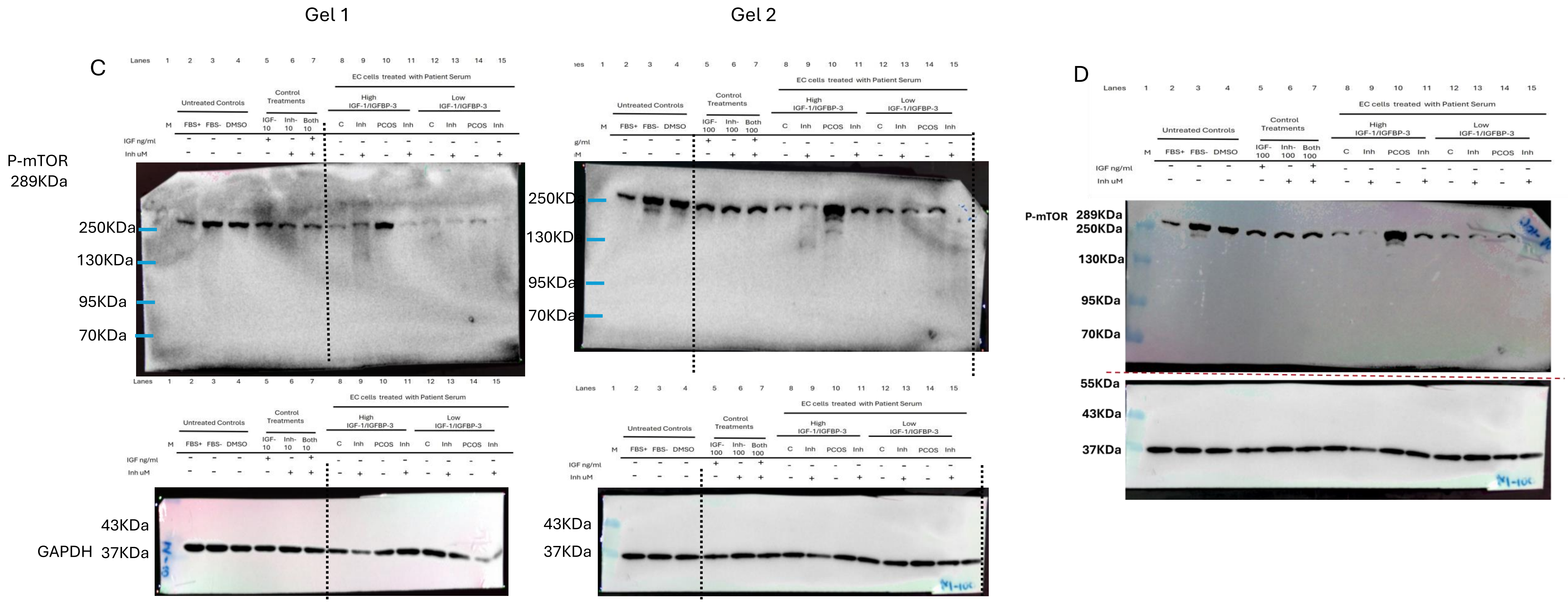

Figure S1 C) original 8% p-mTOR and GAPDH western blots. Dash lines indicate the area From Gel2 cropped and merged with Gel1. D) Full Blot cut at 55KDa with molecular markers. Top half detected for p-mtor and lower half for GAPDH.

Dubai Health - Internal Use
