# Supplementary material for: PCOS Serum Promotes Endometrial Cancer Cell Proliferation with Partial Involvement of IGF-Associated AKT/mTOR Signaling: A Pilot Translational Study with Exploratory Molecular Insights
Source: Int J Mol Sci. 2026 Jul 24;27(15):6594. doi: 10.3390/ijms27156594 (PMC13466320; doi:10.3390/ijms27156594)
Supplement: Supplementary file 1 [file ijms-27-06594-s001.zip › ijms-4309926-supplementary.pdf]

Supplementary Tables

Table S1: List of the 17 cBioPortal datasets included in the integrated endometrial cancer genomic analysis.

No.	Dataset
1	ERBB2-mutant Endometrial cancer (MSK,2024)
2	Endometrial Cancer (MSK, 2018)
3	Endometrial Cancer (MSK, Cancer Discovery 2023)
4	Endometrial Carcinoma (CPTAC, Cell 2020)
5	Endometrial Carcinoma (TCGA, GDC)
6	Endometrial Carcinoma MSI (MSK, Clin Cancer Res 2022)
7	Endometrial Carcinoma cfDNA (MSK, Clin Cancer Res 2022)
8	Uterine Carcinosarcoma (Johns Hopkins, Nat Commun 2014)
9	Uterine Carcinosarcoma (TCGA, Firehose Legacy)
10	Uterine Carcinosarcoma (TCGA, PanCancer Atlas)
11	Uterine Carcinosarcoma/Uterine Malignant Mixed Mullerian Tumor (TCGA, GDC)
12	Uterine Clear Cell Carcinoma (NIH, Cancer 2017)
13	Uterine Corpus Endometrial Carcinoma (TCGA, Firehose Legacy)
14	Uterine Corpus Endometrial Carcinoma (TCGA, Nature 2013)
15	Uterine Corpus Endometrial Carcinoma (TCGA, PanCancer Atlas)
16	Uterine Endometrioid Carcinoma (CPTAC, GDC)
17	Uterine Sarcoma/Mesenchymal (MSK, Clin Cancer Res 2020)

Table S2: List of genes showing widespread co-activation across IGF and downstream pathways. Only statistically significant co-occurring or mutually exclusive gene pairs meeting the predefined significance threshold (Log2 Odds Ratio ≥3 or ≤−3 and q-value <0.01) are shown. Values reported as '>3' indicate gene pairs exceeding the selected reporting threshold rather than exact Log2 Odds Ratio values

A	B	Neither	A Not B	B Not A	Both	Log2 Odds Ratio	p-Value	q-Value	Tendency
IGF1	SOCS2	1592	22	33	16	>3	<0.001	<0.001	Co-occurrence
IGFBP1	IGFBP3	1591	38	19	15	>3	<0.001	<0.001	Co-occurrence
IGF1	IGF1R	3189	20	124	25	>3	<0.001	<0.001	Co-occurrence
IGFBP1	IGF2R	1448	13	162	40	>3	<0.001	<0.001	Co-occurrence
IGFBP2	IGFBP5	1616	28	13	6	>3	<0.001	<0.001	Co-occurrence
IGF1	RAF1	3230	27	83	18	>3	<0.001	<0.001	Co-occurrence
IGF1R	IGFBP3	1515	114	13	21	>3	<0.001	<0.001	Co-occurrence
PIK3CB	RAF1	3114	143	51	50	>3	<0.001	<0.001	Co-occurrence
IGF1R	SOCS2	1508	106	20	29	>3	<0.001	<0.001	Co-occurrence
IGF1R	RAF1	3149	108	60	41	>3	<0.001	<0.001	Co-occurrence
MAP2K2	MAPK3	3155	59	35	13	>3	<0.001	<0.001	Co-occurrence
IGF1	MTOR	3056	17	257	28	>3	<0.001	<0.001	Co-occurrence
PIK3CD	RAF1	3175	82	68	33	>3	<0.001	<0.001	Co-occurrence
RAF1	MAP2K1	3219	83	38	18	>3	<0.001	<0.001	Co-occurrence
RAF1	SOCS2	1555	59	29	20	>3	<0.001	<0.001	Co-occurrence
IGF1	PIK3CD	3214	29	99	16	>3	<0.001	<0.001	Co-occurrence
MAPK3	SOCS1	3188	42	26	6	>3	<0.001	<0.001	Co-occurrence
IGFBP3	IRS1	1529	16	100	18	>3	<0.001	<0.001	Co-occurrence
IGF1R	IGF2	3180	129	29	20	>3	<0.001	<0.001	Co-occurrence
IGF1	BRAF	3206	29	107	16	>3	<0.001	<0.001	Co-occurrence
IGF1	MAPK1	3270	37	43	8	>3	<0.001	<0.001	Co-occurrence
IGF1	IGFBP3	1599	30	26	8	>3	<0.001	<0.001	Co-occurrence
IRS1	IGF2	3193	116	31	18	>3	<0.001	<0.001	Co-occurrence
IGF1R	MAP2K2	3163	121	46	28	>3	<0.001	<0.001	Co-occurrence
IRS1	IGF2R	1413	48	132	70	>3	<0.001	<0.001	Co-occurrence

Dubai Health - Internal Use

IGF1R	PIK3CD	3134	109	75	40	>3	<0.001	<0.001	Co-occurrence
IRS1	RAF1	3156	101	68	33	>3	<0.001	<0.001	Co-occurrence
IGF1	IRS1	3195	29	118	16	>3	<0.001	<0.001	Co-occurrence
IGFBP3	IGFBP4	1583	24	46	10	>3	<0.001	<0.001	Co-occurrence
IRS1	MAP2K1	3187	115	37	19	>3	<0.001	<0.001	Co-occurrence
PIK3CD	MTOR	3017	56	226	59	>3	<0.001	<0.001	Co-occurrence
IGFBP3	MTOR	1440	12	189	22	>3	<0.001	<0.001	Co-occurrence
IGF1R	MAP2K1	3173	129	36	20	>3	<0.001	<0.001	Co-occurrence
AKT2	MAPK1	3179	128	33	18	>3	<0.001	<0.001	Co-occurrence
PIK3CD	MAP2K2	3190	94	53	21	>3	<0.001	<0.001	Co-occurrence
MAP2K1	MAPK3	3166	48	40	8	>3	<0.001	<0.001	Co-occurrence
IGF1	PIK3CB	3139	26	174	19	>3	<0.001	<0.001	Co-occurrence
AKT3	SHC1	1439	69	95	60	>3	<0.001	<0.001	Co-occurrence
IGFBP3	BRAF	1564	22	65	12	>3	<0.001	<0.001	Co-occurrence
IGFBP1	RAF1	1549	35	61	18	>3	<0.001	<0.001	Co-occurrence
IGFBP3	GRB10	1555	21	74	13	>3	<0.001	<0.001	Co-occurrence
IGFBP5	SOCS2	1600	14	44	5	>3	<0.001	<0.001	Co-occurrence
IGFBP3	IGF2	1597	27	32	7	>3	<0.001	<0.001	Co-occurrence
IGF1R	IGFBP2	1511	118	17	17	>3	<0.001	<0.001	Co-occurrence
IGF1R	MAPK3	3082	132	31	17	>3	<0.001	<0.001	Co-occurrence
IGF1R	IRS1	3116	108	93	41	>3	<0.001	<0.001	Co-occurrence
IGFBP3	RAF1	1562	22	67	12	>3	<0.001	<0.001	Co-occurrence
MTOR	SOCS2	1433	181	19	30	>3	<0.001	<0.001	Co-occurrence
IGF1	MAP2K2	3248	36	65	9	>3	<0.001	<0.001	Co-occurrence
RAF1	MAP2K2	3201	83	56	18	>3	<0.001	<0.001	Co-occurrence
IGF1R	MTOR	2994	79	215	70	>3	<0.001	<0.001	Co-occurrence
RAF1	IGF2R	1427	34	157	45	>3	<0.001	<0.001	Co-occurrence
IGFBP3	AKT3	1516	18	113	16	>3	<0.001	<0.001	Co-occurrence
SOCS2	IGF2	1584	40	30	9	>3	<0.001	<0.001	Co-occurrence
IGFALS	SOCS1	1604	34	20	5	>3	<0.001	<0.001	Co-occurrence
IGF1	MAPK3	3175	39	42	6	>3	<0.001	<0.001	Co-occurrence
MAP2K1	IGF2R	1445	16	179	23	>3	<0.001	<0.001	Co-occurrence
IGFBP3	MAPK3	1593	27	36	7	>3	<0.001	<0.001	Co-occurrence
GRB2	SOCS1	1573	65	17	8	>3	<0.001	<0.001	Co-occurrence
IRS1	BRAF	3134	101	90	33	>3	<0.001	<0.001	Co-occurrence
IGFBP3	MAP2K2	1579	25	50	9	>3	<0.001	<0.001	Co-occurrence
GRB10	IGF2R	1422	39	154	48	>3	<0.001	<0.001	Co-occurrence
IGF1	IGF2R	1445	16	180	22	>3	<0.001	<0.001	Co-occurrence
IGFBP1	AKT3	1504	30	106	23	>3	<0.001	<0.001	Co-occurrence
IGF1R	PIK3CB	3066	99	143	50	>3	<0.001	<0.001	Co-occurrence
IGFBP1	IGF2	1580	44	30	9	>3	<0.001	<0.001	Co-occurrence
PIK3CB	PIK3CD	3090	153	75	40	>3	<0.001	<0.001	Co-occurrence
PIK3CA	SOCS2	788	826	4	45	>3	<0.001	<0.001	Co-occurrence
IGF1R	IRS2	3147	123	62	26	>3	<0.001	<0.001	Co-occurrence
IGFBP3	PIK3CD	1550	22	79	12	>3	<0.001	<0.001	Co-occurrence
BRAF	IGF2R	1426	35	160	42	>3	<0.001	<0.001	Co-occurrence
PIK3CD	BRAF	3148	87	95	28	>3	<0.001	<0.001	Co-occurrence
IRS2	IGF2	3231	78	39	10	>3	<0.001	<0.001	Co-occurrence
IGFBP1	PIK3CB	1485	28	125	25	>3	<0.001	<0.001	Co-occurrence
MAPK3	SOCS2	1580	34	40	9	>3	<0.001	<0.001	Co-occurrence
IRS2	PIK3CB	3108	57	162	31	>3	<0.001	<0.001	Co-occurrence
IGFBP1	GRB10	1540	36	70	17	>3	<0.001	<0.001	Co-occurrence
IRS1	MAP2K2	3170	114	54	20	>3	<0.001	<0.001	Co-occurrence
IGFBP2	IRS1	1525	20	104	14	>3	<0.001	<0.001	Co-occurrence
IGF1	IRS2	3234	36	79	9	>3	<0.001	<0.001	Co-occurrence
IGF1	AKT3	3051	24	262	21	>3	<0.001	<0.001	Co-occurrence
IRS2	MAP2K2	3210	74	60	14	>3	<0.001	<0.001	Co-occurrence
MTOR	RAF1	3017	240	56	45	>3	<0.001	<0.001	Co-occurrence
PIK3CD	IGF2	3206	103	37	12	>3	<0.001	<0.001	Co-occurrence
IRS1	MTOR	2997	76	227	58	>3	<0.001	<0.001	Co-occurrence
MAP2K1	SOCS2	1583	31	41	8	>3	<0.001	<0.001	Co-occurrence

AKT3	IGF2R	1396	65	138	64	>3	<0.001	<0.001	Co-occurrence
PIK3CD	SOCS2	1539	75	33	16	>3	<0.001	<0.001	Co-occurrence
IGFBP3	AKT2	1544	22	85	12	>3	<0.001	<0.001	Co-occurrence
IGFBP3	MAPK1	1594	28	35	6	>3	<0.001	<0.001	Co-occurrence
IGF1	AKT2	3180	32	133	13	>3	<0.001	<0.001	Co-occurrence
IRS1	PIK3CD	3137	106	87	28	>3	<0.001	<0.001	Co-occurrence
IGFBP4	MAPK1	1575	47	32	9	>3	<0.001	<0.001	Co-occurrence
IGF1R	BRAF	3118	117	91	32	>3	<0.001	<0.001	Co-occurrence
IGFBP2	MAPK3	1592	28	37	6	>3	<0.001	<0.001	Co-occurrence
IRS2	RAF1	3186	71	84	17	>3	<0.001	<0.001	Co-occurrence
RAF1	IGF2	3218	91	39	10	>3	<0.001	<0.001	Co-occurrence
IGFBP1	MAP2K1	1579	45	31	8	>3	<0.001	<0.001	Co-occurrence
IRS1	MAPK1	3186	121	38	13	>3	<0.001	<0.001	Co-occurrence
IRS2	MAP2K1	3224	78	46	10	>3	<0.001	<0.001	Co-occurrence
PIK3CB	MAPK1	3131	176	34	17	>3	<0.001	<0.001	Co-occurrence
PIK3CB	IGF2R	1380	81	133	69	>3	<0.001	<0.001	Co-occurrence
IGF1	IGFBP4	1577	30	48	8	>3	<0.001	<0.001	Co-occurrence
IGFBP1	IRS2	1563	42	47	11	>3	<0.001	<0.001	Co-occurrence
IGFBP4	GRB10	1536	40	71	16	>3	<0.001	<0.001	Co-occurrence
RAF1	MAPK1	3216	91	41	10	>3	<0.001	<0.001	Co-occurrence
MAP2K2	IGF2R	1432	29	172	30	>3	<0.001	<0.001	Co-occurrence
IGF1R	IGFBP1	1496	114	32	21	>3	<0.001	<0.001	Co-occurrence
GRB2	MAP2K1	1561	63	29	10	>3	<0.001	<0.001	Co-occurrence
IGFBP1	IRS1	1511	34	99	19	>3	<0.001	<0.001	Co-occurrence
GRB10	RAF1	1518	66	58	21	>3	<0.001	<0.001	Co-occurrence
PIK3CB	MTOR	2952	121	213	72	>3	<0.001	<0.001	Co-occurrence
MTOR	IGF2R	1337	124	115	87	>3	<0.001	<0.001	Co-occurrence
PIK3CB	BRAF	3078	157	87	36	>3	<0.001	<0.001	Co-occurrence
IGF1R	IGF2R	1387	74	141	61	>3	<0.001	<0.001	Co-occurrence
IGFBP3	IRS2	1578	27	51	7	>3	<0.001	<0.001	Co-occurrence
RAF1	MAPK3	3124	90	39	9	>3	<0.001	<0.001	Co-occurrence
IGFBP1	MAPK3	1575	45	35	8	3	<0.001	<0.001	Co-occurrence
AKT1	PTEN	1164	110	2038	46	-2.066	<0.001	<0.001	Mutual exclusivity

Table S3: List of Key Co-activated Gene Pairs.

Pathway Node	Top Co-occurring Partners	Implication
IGF1R	*IGF1*, *IRS1/2*, *MAP2K2*, *MTOR*	Central signaling hub integrating both upstream IGF input and downstream pathway propagation
PIK3CB/PIK3CD	*RAF1*, *MTOR*, *MAPK* genes	Parallel activation of PI3K and MAPK suggests extensive pathway crosstalk
IGFBPs	*IGF2R*, *AKT3*, *GRB10*, *IRS1*	May regulate ligand availability and downstream amplification
MAPK Cascade	*RAF1*, *MAP2K1*, *MAP2K2*, *MAPK3*	Suggests intact and active MAPK module downstream of IGF1R
SOCS1/SOCS2	*IGF1*, *MTOR*, *MAPK1*	May represent feedback inhibition attempts within hyperactive signaling networks

Dubai Health - Internal Use
